# Prevalence of antibiotic resistant *Escherichia coli* isolates from fecal samples of food handlers in Qatar

**DOI:** 10.1186/s13756-018-0369-2

**Published:** 2018-06-26

**Authors:** Nahla O. Eltai, Hadi M. Yassine, Asmaa A. Al Thani, Marwan A. Abu Madi, Ahmed Ismail, Emad Ibrahim, Walid Q. Alali

**Affiliations:** 1 Biomedical Research Center, Qatar University, Doha, Qatar; 2 College of Health Sciences, Qatar University, Doha, Qatar; 3 Laboratory Services, Medical Commission, Ministry of Public Health, Doha, Qatar; 4 Department of Lab Medicine and Pathology, Hamad Medical Hospital, Doha, Qatar; 5 College of Public Health, Hamad Bin Khalifa University, Doha, Qatar; 6 Present Address: Department of Veterinary Medicine, College of Food and Agriculture, United Arab Emirates University, Alain, United Arab Emirates

**Keywords:** Food handlers, *E. coli*, Antibiotic resistance, Multi-drug resistant, ESBL

## Abstract

**Background:**

It is well documented that food handlers harbor and shed enteric foodborne pathogens causing foodborne disease outbreaks. However, little known on enteric antibiotic resistant (AR) bacteria carriage in food handlers. The objective of this study was to establish a baseline prevalence of fecal AR *E. coli* among food handlers in Qatar.

**Methods:**

Fecal samples were collected from 456 migrant food handlers of different nationalities arriving in Qatar on a work permit between January 2015 and December 2016. These samples (25 g each) were collected based on the availability and examination schedule at the Medical Commission facility from those consented to participate. Isolated *E. coli* bacteria were tested for antibiotic susceptibility against nine antibiotics using the E-test method and Double Disc Synergy Test (DDST) for extended-spectrum beta-lactamase (ESBL) production.

**Results:**

From the 78 *E. coli* positive samples (17.1%, *n* = 456), 60% of the isolates were resistant to at least one antibiotic, whereas, 27% were multi-drug resistant (MDR). Seven isolates (9%, *n* = 78) were ESBL producers of which five were MDR. Individual AR *E. coli* frequencies to the nine antibiotics were not significantly (*P* > 0.05) different by nationality.

**Conclusions:**

Based on our findings, we revealed that individual resistant *E. coli* and MDR resistant *E. coli* were common in fecal samples of food handlers in Qatar. This may indicate that food handlers can potentially contaminate foods with AR *E. coli*, a possible public health concern.

## Background

Antibiotic resistance (AR) continues to pose a great threat to public health in both developed and developing countries [1, 2]. Infections caused by AR bacteria, especially multi-drug resistant organisms, can lead to serious health problems such as prolonged hospitalization, treatment failure and deaths [1, 2]. A worrying increase in multi-drug resistant phenotypes in enterobacteriaceae including *E. coli* and particularly to third-generation cephalosporins as well as to colistin (last resort antibiotic used to treat carbapenem-resistant enterobacteriaceae) has been reported [3–8]. The emergence, development, and spread of AR bacteria is a complex issue that involves multiple factors such as the use and misuse of antibiotics, poor infection control practices, inadequate sanitary conditions, and inappropriate food-handling practices [9–12]. Accordingly, food handlers with poor personal hygiene and inadequate food safety knowledge working in food service establishments can be a potential source for food contamination that can lead to foodborne disease and outbreaks [13, 14]. Likewise, food handlers who harbor AR bacteria in their gastrointestinal tract may contaminate foods which is considered as a potential route AR bacteria transmission to consumers [15].

Food handlers have been linked to a large number of foodborne outbreaks worldwide. A total of 816 foodborne outbreaks with 80,682 foodborne illnesses occurred between 1927 and 2006 were linked to food handlers (Greig et al., 2007). Likewise, The Centre for Disease Control and Prevention (CDC) revealed that 52% of 9040 foodborne disease outbreaks reported between 1998 and 2004 were associated with food services (i.e., restaurants, cafeterias, and hotels) [16]. Approximately, bacterial agents caused 13% of these outbreaks. The majority of foodborne outbreaks associated with food services were associated with unsanitary conditions of the food handlers [17, 18].

*Escherichia coli* bacteria are very abundant in the human gastrointestinal tract and can serve as carriers/reservoirs of AR genes. These bacteria are also able to share AR genetic material with their fellow bacteria of the same and/or different species [19, 20]. The incidence of AR bacteria are rapidly increasing in humans in Qatar (Personal communication with the Microbiological diagnostic Laboratory of Hamad Medical Corporation, Doha, Qatar). A hospital-based AR bacteria surveillance system exists in Qatar to monitor the incidence of a number of AR bacteria from outpatient and in-patient clinics (Hamad Medical Corporation, Doha, Qatar). However, there is no available information on the AR enteric bacteria such as *E. coli* from non-clinical samples [21–23].

There is a large and continuous influx of migrant labor workers in Qatar; largely from south-east Asian countries [24]. A group of those migrant workers comes to Qatar on a work permit designated for jobs within the food industry such as food handling and service. Many of the food handlers have limited knowledge of risks associated with food contamination. Nonetheless, they may harbor and shed AR commensal *E. coli* during handling of food leading to food contamination. Hence, resistant bacteria can spread to the public through consumption of contaminated food. Research studies have shown that multi-drug resistant *E. coli* can be present in ‘healthy’ individual in community settings [25–29] even with the absence of antibiotic use [30]. Furthermore, several studies have revealed that AR *E. coli* from food source can spread to humans, colonize human gut, and potentially cause infections such as urinary tract infections [31–35].

There is currently limited information on AR phenotypes in commensal enteric organisms, from non-clinical human populations in Qatar. A first step to assess the AR risk associated with food handlers is to obtain a baseline prevalence of AR *E. coli* (an indicator organism) in fecal samples collected from this cohort. Therefore, the objective of this study was to determine the prevalence of the AR *E. coli* isolates from fecal samples of migrant food handlers in Qatar.

## Methods

### Study population and sampling scheme

All migrant workers including food handlers arriving to Qatar on work permit have to go through a mandatory medical screening at the Medical Commission, Department of Ministry of Public Health, Doha, Qatar [36]. Individual fecal samples were collected randomly from apparently healthy food handlers within one week of arrival to Qatar. The collection of the fecal samples was conducted between January 2015 and December 2016 as part of another research study that focused on presence of enteric parasites (Abu Madi et al., College of Health Sciences, Qatar University). All participants were provided with informed consent form explaining the purpose of the study and clearly reassured no obligation to participate; moreover, they have been comforted that all collected data will be treated with high confidentiality. The fecal samples (25 g each) were collected based on the availability and examination schedule at the Medical Commission Department from those consented to participate. Nationality information was obtained for each food handler. Portions of the fecal samples were used for the parasite analysis, while the leftover samples (~ 10 g) were stored in the freezer at − 20 °C. For this study, all available frozen fecal samples (*n* = 456) representing 456 food handlers collected over two year period (January 2015 to December 2016) were used i. These samples were utilized for *E. coli* isolation and antibiotic susceptibility testing. The food handlers included in this study were all males between the ages of 20 and 35 year old. There were 139, 118, 110, 40, 25, 16, 5, and 3 fecal samples from food handlers that originated from India, Bangladesh, Nepal, Philippine, Sir Lanka, Indonesia, Kenya, and Thailand, respectively. There were no reports of acute or chronic infections in the food handlers. No additional identifiable information were available for this study. Moreover, historical antibiotic use data associated with the study subjects were not available. The Institutional Review Board (IRB) of Ministry of Public Health (Doha, Qatar) approved this study in June 2017.

### *E. coli* isolation and identification

To isolate *E. coli*, fecal samples were thawed at room temperature (25 °C), streaked directly onto a selective medium CHROMagar™ *E. coli* plates (Difco, Becton Dickenson, Sparks, MD) using sterile cotton-tipped swabs, and then incubated at 37 °C for 18–24 h. A typical *E. coli* colony (green color with smooth surface) was randomly selected and subsequently streaked onto 5% sheep blood agar (Difco, BD) plates, then incubated at 37 °C for 18–24 h to obtain pure single colonies. For confirmation, suspected *E. coli* colonies were transferred onto MacConkey agar (Difco, BD) plates and incubated (37 °C, 18–24 h). Thereafter, colonies were streaked onto fresh blood agar plates and incubated (37 °C, 18–24 h) for isolation. Up to three colonies from the blood agar were tested for lactose fermentation using the Indole spot test (Remel, Thermoscientific, Lenexa, KS, USA). *Escherichia coli* isolates were transferred to Cryovial tubes (TechnicalService, Lancashire, U.K.) and stored at − 80 °C until further analysis.

### Antibiotic susceptibility testing of *E. coli* isolates

Frozen *E. coli* isolates were revived from the Cryovials on blood agar plates. A single *E. coli* isolate was randomly selected from the blood agar plate and suspended in 0.85% saline (Difco, BD) to achieve an inoculum equivalent to 0.5 McFarland standard as measured by DensiCHEK™ Plus spectrophotometer (Biomérieux, Marcy, France). The suspension was swabbed on a Mueller-Hinton agar plate (MH; Difco, BD) using a sterile cotton-tip and allowed to dry completely at room temperature. An antibiotic susceptibility test strip (E-test strip, BioMerieux) was then applied to the MH agar surface with sterile forceps and the plate was incubated at 37 °C for 18–24 h. Thereafter, the minimum inhibitory concentration (MIC) was read directly from the test strip where the elliptical zone of inhibition intersected with the MIC scale on the strip, according to the manufacturer’s instructions. The MIC values were interpreted according to the Clinical & Laboratory Standards Institute (CLSI) guidelines [37]. *Escherichia coli* strains ATCC® 25,922 and 35,218 were used as controls. The nine antibiotics utilized for antibiotic susceptibility testing of *E. coli* isolates and their breakpoints are shown in Table 1.

**Table 1:** Minimum inhibitory concentration (MIC) range and interpretation for antibiotics used to test susceptibility of *E. coli* isolates (*n* = 78) from food handlers’ fecal samples

Antibiotic	Abbreviation	MIC range tested (μg/mL)	MIC Interpretive Standard (μg/mL)^a^ SIR		
Penicillin & Penicillin β-lactamase inhibitor combinations					
Ampicillin	AM	0.016–256	≤8	16	≥32
Amoxicillin/Clavulanic acid	XL	0.016–256	≤8/4	16/8	≥32/16
Aminoglycosides					
Gentamicin	GM	0.016–256	≤4	8	≥16
Quinolone					
Ciprofloxacin	CI	0.002–32	≤0.006	0.12–0.5	≥1
Chloramphenicol					
Chloramphenicol	CL	0.016–256	≤8	16	≥32
Tetracycline					
Tetracycline	TC	0.016–256	≤4	8	≥16
Folate pathway inhibitors					
Trimethoprim	TR	0.002–32	≤8	–	≥16
Sulphamethoxazole	SX	0.064–1024	≤258		≥512
Cephalosporin					
Ceftriaxone	TX	0.016–256	≤1	2	≥4

^a^ The MIC values interpretation into sensitive (S), intermediate (I), and resistant (R) was according to the Clinical & Laboratory Standards Institute (CLSI) guidelines

### Double disc synergy test (DDST)

**Fig. 1:**
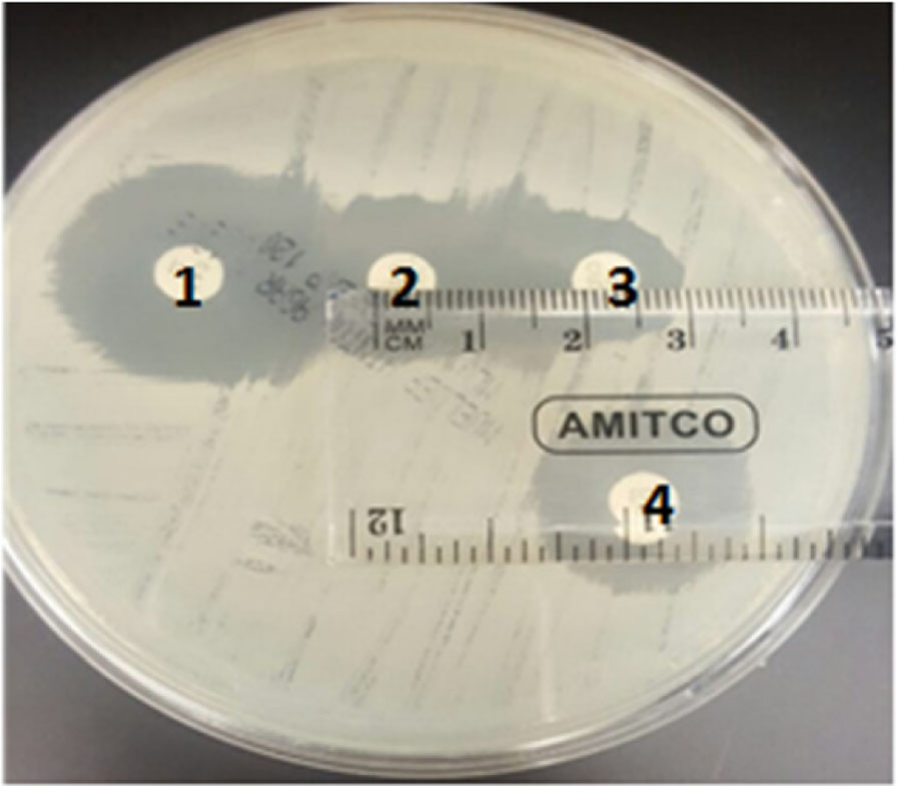
Double Disc Synergy Test (DDST) confirming ESBL-Producing *E. coli* isolates from fecal samples of food handlers (Antibiotic discs and concentrations used were as follows: 1. Ceftazidime (30 μg); 2. Amoxicillin/Clavulanic acid (20/10 μg); 3. Ceftriaxone (30 μg); and 4. cefoxitin (30 μg))

All *E. coli* isolates that showed resistant to 3rd generation cephalosporins (i.e., ceftriaxone in this study) with MIC ≥4 based on the E-test strip results were further screened for extended-spectrum beta-lactamase (ESBL) production via the Double Disc Synergy Test (DDST) as described elsewhere [37]. Briefly, *E. coli* isolates from blood agar plate were suspended in 0.85% saline to achieve an inoculum equivalent to 0.5 McFarland standard as described earlier. The suspension was then plated on an MH agar plate and a disc containing amoxicillin-clavulanate (20/10 μg, BD- Sensi Disc™) was placed in the center of the plate. Next, one disc of ceftriaxone (30 μg, BD- Sensi Disc™) and another disk of ceftazidime (30 μg, BD- Sensi Disc™) were placed 15 mm apart from the edge of amoxicillin-clavulanate disc as shown in Fig. 1. The cefoxitin (30 μg, BD- Sensi Disc™) disc was placed in any available space remaining on the plate [37, 38]. The MH plates with the four antibiotic discs were incubated at 37 °C for 18–24 h. Any *E. coli* isolate that showed an increase in the zone of inhibition around either ceftazidime or ceftriaxone (i.e., > 5 mm towards the disc of amoxicillin-clavulanate) together with susceptibility to cefoxitin was interpreted as positive for the ESBL production. *Escherichia coli* ATCC® 51,446 and 25,922 were used as control strains for positive and negative ESBL production, respectively.

### Data analysis

The antibiotic resistance outcomes (resistance or susceptible [i.e., binary]) and multidrug-resistant (MDR) isolates (those resistant to three or more antimicrobial classes) were cross tabulated with nationality using a Fisher’s exact test or 2-by-n likelihood ratio chi-square test, as appropriate, in STATA 14.1 software (Stata Corp., College Station, TX).

## Results

From the 456 food handler fecal samples tested, 78 (17.1%) were positive for *E. coli*. In general, isolates were frequently resistant to sulfamethoxazole (33.3%), followed by ampicillin (32.1%), trimethoprim (30.8%), and tetracycline (25.6%). The *E. coli* isolates were less resistant to ciprofloxacin (14.2%), ceftriaxone (9%), and chloramphenicol (3.9%).

**Fig. 2:**
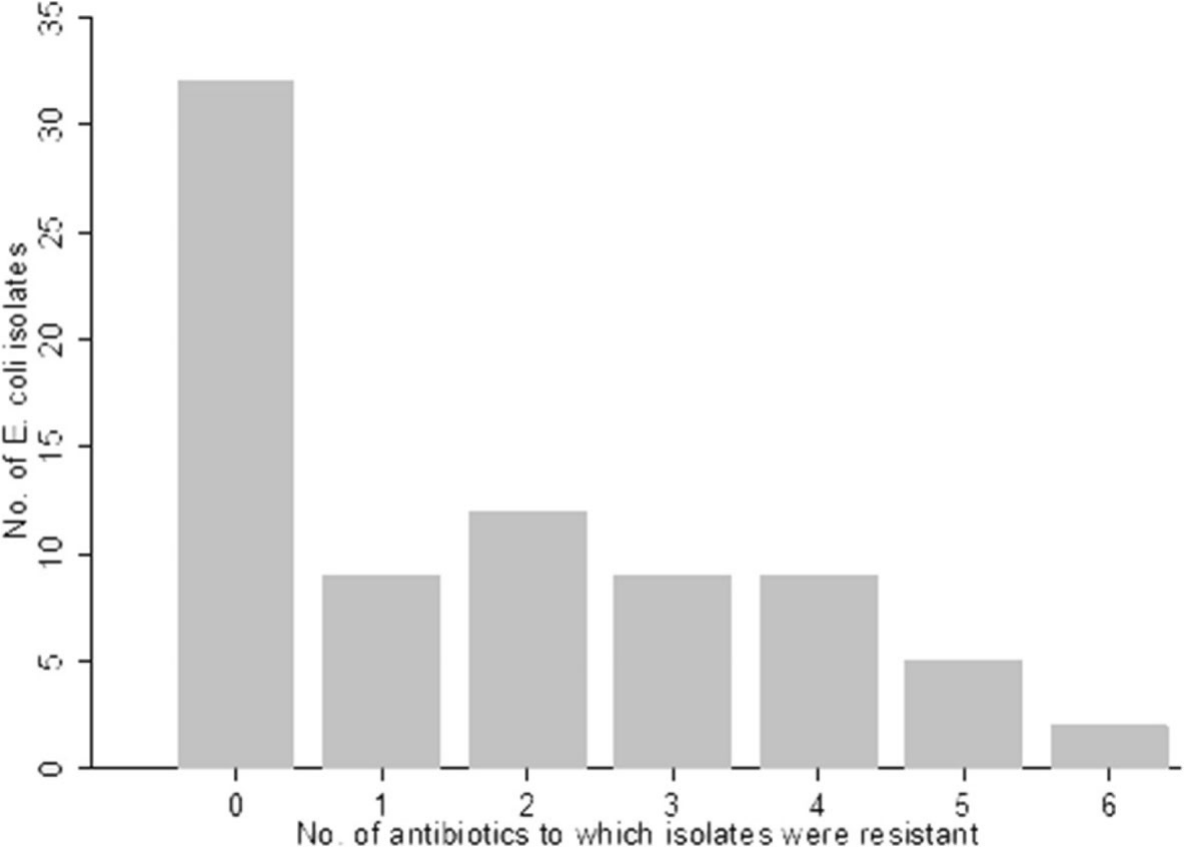
Frequency bar chart illustrating the distribution of phenotypic antibiotic resistance to up to six antibiotics among *E. coli* isolates (*n* = 79) from food handlers’ fecal samples in Qatar

The distribution of *E. coli* isolates by nationality is shown in Table 2. The individual antibiotic resistant *E. coli* frequencies were not significantly (*P* > 0.05) different by nationality. The antibiotic resistance profiles among the 78 *E. coli* isolates are summarized in Table 3. The distribution of phenotypic antibiotic resistance to the 9 antibiotics is shown in Fig.2. Approximately, 59% of the isolates were resistant to at least one antibiotic, whereas 11.5, 15.4%, 11.5, 11.5, 6.4 and 2.6% isolates showed resistance to one, two, three, four, five and six antibiotics, respectively (Fig. 2). Twenty-seven percent (*n* = 78) of all *E. coli* isolates were multi drug resistant (MDR). Moreover, 9% (seven) of the *E. coli* isolates (*n* = 78) were ESBL producers; of which five were MDR isolates.

**Table 2:** Distribution of fecal *E. coli* isolates (*n* = 78) by food handler’s nationality

Nationality	Frequency	Percentage ^a^
Indian	30	38.5
Bangladesh	19	24.4
Nepalese	13	16.7
Philippine	8	10.3
Indonesian	3	3.8
Kenyan	2	2.6
Siri Lankan	2	2.6
Thai	1	1.3

^a^ Percentages of antibiotic resistant *E. coli* isolates by nationality were not significantly different (*P* > 0.05) based on chi-square test in STATA 14.1 software

**Table 3:** Phenotypic resistant profiles of *E. coli* isolates from food handlers’ fecal samples (*n* = 78)

Resistant phenotype	Frequency	Percentage ^c^
No resistance	32	41
Resistant to only one antibiotic	9	11.5
Trimethoprim; Sulfamethoxazole	5	6.4
Ampicillin; Trimethoprim; Sulfamethoxazole	4	5.12
^a b^ Ciprofloxacin; Ampicillin; Ceftriaxone; Trimethoprim; Sulfamethoxazole; Tetracycline	2	2.6
^a^ Trimethoprim; Sulfamethoxazole; Tetracycline; Ampicillin	3	3.8
^a^ Tetracycline; Ciprofloxacin; Trimethoprim; Sulfamethoxazole	3	3.8
^a^ Ampicillin; Trimethoprim; Sulfamethoxazole; Tetracycline; Ciprofloxacin	2	2.6
^a b^ Ampicillin; Trimethoprim; Ceftriaxone; Tetracycline; Ciprofloxacin	1	1.3
^a^ Chloramphenicol; Sulphamethoxazole; Tetracycline	2	2.6
^a^ Ampicillin; Trimethoprim; Tetracycline	2	2.6
^b^ Ampicillin; Ceftriaxone	2	2.6
Ampicillin; Tetracycline	2	2.6
Ampicillin; Trimethoprim	1	1.3
Trimethoprim; Tetracycline	1	1.3
Trimethoprim; Sulfamexazole; Ciprofloxacin	1	1.3
Ampicillin; trimethoprim; Sulfamethxazole; Amoxcicillin-Clavulanic acid	1	1.3
Ampicillin; Sulfamethoxazole	1	1.3
^a b^ Ampicillin; Sulfamethoxazole; Ceftriaxone; Ciprofloxacin	1	1.3
^a^ Ampicillin; Trimethoprim, Sulfamethoxazole; Ciprofloxacin	1	1.3
^a b^ Ampicillin; Tetracycline; Trimethoprim; Sulfamethoxazole; Ceftriaxone	1	1.3
Ampicillin; Chloramphenicol; Tetracycline; Trimethoprim; Sulfamethoxazole	1	1.3

*MDR* multi drug resistant, *ESBL* extended spectrum beta lactamase producer

^a^MDR (*n* = 19)

^b^ESBL isolates; ESBL + MDR (21)

^c^Percentages of phenotypic resistant profiles of *E. coli* were significantly different (*P* < 0.05) based on chi-square test in STATA 14.1 software

## Discussion

The incidence of AR bacteria in public hospital and clinics is monitored by the Hamad Medical Corporation (HMC), which is Qatar’s main health care provider. According to recent reports by HMC, a notable increase in the incidence of AR bacteria in individuals attending out- and in-patient facilities has been observed [39, 40]. However, there are limited AR bacteria data on individuals in non-hospital (i.e., community) settings such as food handlers. To the best of our knowledge, this is the first study to investigate the prevalence of AR enteric *E. coli* in apparently healthy food handlers. In this pilot study, we reported remarkable percentages of *E. coli* isolates resistant to at least one antibiotic (i.e., 59%) with 27% being MDR and 9% being ESBL producing isolates. The majority of the food handlers in this study had limited knowledge of risk associated with food contamination (personal communication with the Medical Commission staff). This group may harbor and shed AR enteric *E. coli* bacteria that can contaminate food prepared for and consumed by people in Qatar. Thus, these findings suggest that resistant *E. coli* isolates are present in the community; hence, aggravating the public health concerns regarding the spread of AR bacteria. A one-health approach ought to be considered to address this food safety and public health problem.

There are over 1300 food service establishments (restaurant and hotels) in Qatar that employ more than 50,000 workers with the majority being food handlers [41]. Food handlers have been linked to large number of foodborne outbreaks worldwide. The majority of foodborne outbreaks associated with food services are due to unsanitary conditions of the food handlers [17, 18] . Therefore, they can easily contaminate the food they handle with pathogens and non-pathogens. In agreement with our findings, several studies have shown that multi-drug resistant *E. coli* including commensal isolates harboring ESBLs can be present in healthy individuals [42–44]. Garedew-Kifelew L, Wondafrash N and Feleke A [45] examined stool specimens of food handlers from Ethiopia and revealed that 3.1% of the subjects were infected with AR *Salmonella*. Similarly, Greig JD, Todd EC, Bartleson CA and Michaels BS [46] concluded that most of the foodborne outbreaks were associated with food handlers who were asymptomatic carrier and excrete the pathogen unknowingly while working, or they were sick but continue to prepare food.

One of the limitations of this study was the low recovery percentage of *E. coli* from fecal sample (~ 17%) which could be due to the long storage of fecal samples in − 20 °C over 2 year period and the direct plating method of the samples on the selective medium. Another limitation was that the sample collection was from the participants conducted as part of another research study that focused on presence of enteric parasites; hence, we had no control over the sampling scheme. Moreover, the results regarding the AR *E. coli* by nationality need to be interpreted with caution as the number of isolates from some nationalities was low.

## Conclusions

In conclusion, food handlers that are apparently healthy individuals could harbor and shed AR enteric *E. coli,* posing a significant public health risk to the general population in Qatar. This may occur via dissemination of AR *E. coli* through contaminated food. Despite the potential effect encountered by the transmission of the opportunistic bacteria through food handlers, this is the first pilot study to screen the AR *E. coli* among apparently healthy food handlers. More research studies are needed to longitudinally follow AR commensal and pathogenic bacteria in food handlers and the relationship with unique and common strains found in hospital settings. A better infection control and improved food safety plans could reduce the risk of AR bacteria contamination of food in Qatar. This could be through delivering proper training to food handlers in order to prevent the contamination of foods. In addition, one-health approach with collaboration between food safety, public health, and veterinary service entities can further address this concern.

## Abbreviations

AR: Antibiotic resistant
DDST: Double Disc Synergy Test
ESBL: Extended-spectrum beta-lactamase
MDR: Multi-drug resistant
